# Appropriate whole genome amplification and pathogenic loci detection can improve the accuracy of preimplantation genetic diagnosis for deletional α-thalassemia

**DOI:** 10.3389/fendo.2023.1176063

**Published:** 2024-03-08

**Authors:** Yueyun Lan, Hong Zhou, Sheng He, Jinhui Shu, Lifang Liang, Hongwei Wei, Jingsi Luo, Caizhu Wang, Xin Zhao, Qingming Qiu, Peng Huang

**Affiliations:** ^1^ Maternal and Child Health Hospital of Guangxi Zhuang Autonomous Region, Nanning, China; ^2^ Birth Defects Prevention and Control Institute of Guangxi Zhuang Autonomous Region, Nanning, China; ^3^ Guangxi Key Laboratory of Reproductive Health and Birth Defect Prevention, Nanning, China; ^4^ Genetic and Metabolic Central Laboratory of Maternal and Child Health Hospital of Guangxi Zhuang Autonomous Region, Nanning, China; ^5^ Guangxi Key Laboratory of Precision Medicine for Genetic Diseases, Nanning, China; ^6^ Guangxi Key Laboratory of Birth Defects Research and Prevention, Nanning, China

**Keywords:** deletional α-thalassemia, preimplantation genetic testing, whole genome amplification, SNP haplotype analysis, next generation sequencing

## Abstract

**Objective:**

To improve the accuracy of preimplantation genetic testing (PGT) in deletional α-thalassemia patients.

**Design:**

Article.

**Patient(s):**

fifty-two deletional α-thalassemia couples.

**Intervention(s):**

Whole genome amplification (WGA), Next-generation sequencing (NGS) and PCR mutation loci detection.

**Main outcome measures:**

WGA, Single nucleotide polymorphism (SNP) and PCR mutation loci detection results; Analysis of embryo chromosome copy number variation (CNV).

**Results:**

Multiple Displacement Amplification (MDA) and Multiple Annealing and Looping–Based Amplification Cycles (MALBAC) methods for PGT for deletional α-thalassemia. Blastocyst biopsy samples (n = 253) were obtained from 52 deletional α-thalassemia couples. The results of the comparison of experimental data between groups MALBAC and MDA are as follows: (i) The average allele drop-out (ADO) rate, MALBAC *vs.* MDA = 2.27% ± 3.57% *vs.* 0.97% ± 1.4%, *P*=0.451); (ii) WGA success rate, MALBAC *vs.* MDA = 98.61% *vs.* 98.89%, *P*=0.851; (iii) SNP haplotype success rate, MALBAC *vs.* MDA = 94.44% *vs.* 96.68%, *P*=0.409; (iv) The result of SNP haplotype analysis is consistent with that of Gap-PCR/Sanger sequencing results, MALBAC *vs.* MDA = 36(36/72, 50%) *vs.* 151(151/181, 83.43%), *P*=0; (v) Valid SNP loci, MALBAC *vs.* MDA = 30 ± 9 *vs.* 34 ± 10, *P*=0.02; (vi) The mean CV values, MALBAC *vs.* MDA = 0.12 ± 0.263 *vs.* 0.09 ± 0.40, *P*=0.916; (vii) The average number of raw reads, MALBAC *vs.* MDA =3244259 ± 999124 *vs.* 3713146 ± 1028721, *P*=0; (viii) The coverage of genome (%), MALBAC *vs.* MDA = 5.02 ± 1.09 *vs.* 5.55 ± 1.49, *P*=0.008.

**Conclusions:**

Our findings indicate that MDA is superior to MALBAC for PGT of deletional α-thalassemia. Furthermore, SNP haplotype analysis combined with PCR loci detection can improve the accuracy and detection rate of deletional α-thalassemia.

## 1 Introduction

Thalassemia is one of the most common monogenic diseases in the clinic and comprises a group of hereditary anemias caused by reduced or even absence of one or more globin chains synthesis disorders due to the abnormal globin gene (1). Its symptoms can range from asymptomatic to transfusion-dependent (2). α-Thalassemia is an autosomal recessive disorder cause by deletion or single nucleotide variants (SNVs) in the α-1 and α-2 genes (cis tandem) of the α-like globin gene clusters located at 16p13.3 (chr16: 199800–233300) (3), which result in insufficient synthesis of α-globin peptide chains. α^0^ -Thalassemia is defined as a deletion or abnormality in one alpha gene on each chromosome, while α^+^ thalassemia is defined as a deletion or abnormal in only one α gene on one chromosome. Southeast Asia deletion α^0^-thalassemia (–^SEA^), 3.7-kb deletion (-α^3.7^) and 4.2-kb deletion (-α^4.2^) are the three most common homozygous mutations of deletional α-thalassemia in China (4). In addition, α-1 or α-2 gene SNVs cause non-deletional α-thalassemia (α^+^). The common α^+^ genes are Hb constant Spring (α^CS^α), Hb Westmead (α^WS^α) and Hb Quang Sze (α^QS^α). The gene frequencies of α^CS^α, α^WS^α and α^QS^α recorded at 0.24%, 0.26% and 0.06%, respectively (4). Silent carriers of α-thalassemia with only one gene mutation are asymptomatic and require no treatment. Mild α-thalassemia includes α^0^ -thalassemia heterozygotes (–/αα), α^+^-thalassemia homozygotes (α/-α) or double heterozygotes (-α/α^T^α). These individuals are asymptomatic carriers with no obvious clinical symptoms. α^0^-Thalassemia, is combined with a milder form, α^+^-thalassemia, it can lead to the Hemoglobin H (Hb H) disease (5). Due to the instability of this disorder, affected individuals have increased hemolysis and a mild-to-moderate anemia with marked microcytosis and hypochromia (6). Homozygous α^0^-thalassemia (–^SEA^/–^SEA^) causes Hb Bart’s hydrops fetalis syndrome, which usually results in death either in late gestation or within several minutes of delivery. Hb Bart’s hydrops fetalis syndrome is the most severe form of α-thalassemia. Due to the deletion of all four alpha globin genes on chromosome 16, the alpha globin chain is completely lacking, and the γ globin chain itself polymerized into the tetramer γ4 (7). The extremely high affinity of this form of hemoglobin for oxygen causes tissue hypoxia, resulting in fetal edema and premature birth or stillbirth (8, 9).

Pre-implantation genetic testing (PGT) refers to the genetic testing of pre-implantation embryos in assisted reproduction and the selection of embryos without pathogenic mutations for uterine implantation to avoid familial genetic diseases. PGT can be performed for monogenic disorders or single gene defects (PGT-M), for chromosomal structural rearrangements (PGT-SR), and for aneuploidy detection (PGT-A) (10). PGT-M refers to the identification of nuclear DNA pathogenic variant(s) of a single gene associated with disease for which the pathogenic locus has been identified (11, 12). Such variants have an autosomal dominant, autosomal recessive or X-linked transmission pattern, and the disease-causing locus has been clearly identified in the offspring of affected individuals. If the prospective parents are both carriers of the *HBA* gene, the chances of giving birth to a child with severe α-thalassemia are one in four (2). Techniques for preventing the birth of children with moderate-to-severe thalassemia include prenatal and PGT (13). However, compared with traditional prenatal diagnosis, PGT offers the advantage of avoiding the physical and psychological harm to pregnant women caused by termination of the pregnancy (13). Therefore, PGT technology can be used to select genetically normal embryos (14).

Currently, there are no effective medical treatments for Hb Bart’s hydrops fetalis syndrome or Hb H disease, and scientific and technological advances is required to prevent such birth defects. Thus, to prevent the transmission of *HBA* mutations, PGT can be considered for couples at high risk of having offspring with Hb Bart’s fetal edema syndrome or Hb H disease after genetic counseling. PGT technology, including whole genome amplification (WGA), single nucleotide polymorphism (SNP) haplotype analysis or next-generation sequencing (NGS), represents a major improvement in that some related preclinical examinations can be omitted, which reduces the workload of the laboratory and the waiting time for couples. Multiple Displacement Amplification (MDA) and Multiple Annealing and Looping–Based Amplification Cycles (MALBAC) methods are commonly used for WGA. In this study, we evaluated the MDA and MALBAC methods amplified blastocyst trophoblastic ectodermal cells to test for deletional α-thalassemia. SNP haplotype analysis combined with PCR mutation loci detection were used to diagnose whether the embryo carried *HBA* gene deletional mutation.

## 2 Materials and methods

### 2.1 Patients

This study was conducted according to principles of the Helsinki Declaration and was approved by the Institutional Review Board (IRB) of Maternal and Child Health Hospital of Guangxi Zhuang Autonomous Region (No. [2021-5] 4). A total of 52 couples with both individuals carrying deletional mutations in the *HBA* gene received PGT treatment after being evaluated by geneticists and infertility specialists at the reproductive center of our institution between July 1, 2020 and May 31, 2022. Each couple provided written informed consent before the PGT cycle. Karyotype analyses with conventional G-banding demonstrated that the karyotypes of these couples were normal. None of the couples enrolled had any underlying diseases. However, 22 out of 52 (42.31%, 22/52) deletional α-thalassemia couples had conceived a fetus with Hb H disease or Hemoglobin Bart’s hydrops fetalis syndrome. In the MALBAC group, there were 16 cases of deletional α-thalassemia. In the MDA group, there were 36 cases of deletional α-thalassemia, with –^SEA^/α^WS^α genotype identified in the spouses in two of the couples.

### 2.2 Pedigree analysis

#### 2.2.1 Selection of appropriate detection methods for *HBA* gene mutations

To improve the accuracy of pedigree verification, two different detection methods were used to verify whether the samples carrying *HBA* gene mutations. For deletional α-thalassemia, we performed Gap-PCR and SNP haplotype analyses to identify mutation loci. Sanger sequencing and SNP haplotype analysis were used to identify α-thalassemia non-deletion mutation loci.

#### 2.2.2 Mutation loci detection of *HBA* gene

(i) Genomic DNA was isolated from peripheral blood lymphocytes using LabAid DNA kit (Zeesan Biotech Co., Ltd, Xiamen, China). (ii) Gap-PCR: The deletion thalassemia assay kit (Yikon Genomics, Shanghai, China) was used to detect the deletion range and break point of –^SEA^, -α^4.2^, and -α^3.7^. Deletion region external primers and standard internal control primers were designed. The sequence of primers is shown in **Supplementary Materials Table 1**. Gap-PCR was performed according to the manufacturer’s instructions with the following PCR reaction system: 2× Goldstar Master Mix 10 µL, mutation site PCR primer 1 μL, DNA template X μL (30 ng), ddH_2_O supplementation to 20 µL system. The PCR reaction conditions were as follows: (i) –^SEA^/αα, pre-denaturation at 95°C for 10 min; 95°C for 30 s, 55°C for 30 s, 72°C for 45 s (35 cycles); extension at 72°C for 5 min, 8°C, forever; (ii) -α^3.7^/αα, pre-denaturation at 95°C for 10 min; 97°C for 45 s, 65°C for 90 s, 72°C for 180 s (35 cycles); extension at 72°C for 5 min, 8°C, forever; (iii) -α^4.2^/αα, pre-denaturation at 95°C for 10 min; 95°C for 45 s, 61°C for 90 s, 72°C for 180 s (35 cycles); extension at 72°C for 5 min, 8°C, forever. (iv) Sanger sequencing: α^WS^α was detected using Sanger sequencing. The PCR mixture consisting of 1 μL 0.4 nM mutation point specific primer, 10 μL 2× GoldStar Best MasterMix (CWBIO, China), 30 ng purified WGA product, and 9-X ddH_2_O added to achieve a total volume of 20 μL. The PCR cycling conditions were as follows: pre-denaturation at 94°C for 5 min; 94°C for 30 s, 68°C for 2 min (40 cycles); extension at 72°C for 10 min, 8°C, forever. For purification, 1 μL of the prepared digestion solution (0.5 μL alkaline phosphatase: 0.5 μL exonuclease І) was added to the PCR reaction solution and mixture was incubated at 37°C for 6 min followed by 80°C for 15 min. The purified PCR product was then stored at 4°C. Sanger sequencing was performed using the SEQ Mix according to ABI PRISM^®^ BigDye^®^ Terminator v3.1 Cycle Sequencing Kit. Briefly, 3.5 μL Mix, 1 μL primer, and 1 μL PCR product were mixed a 96-well plate according to the sequencing reaction protocol. The SEQ procedure was as follows: pre-denaturation at 96°C for 2 min; 96°C for 10 s, 55°C for 5 s, 60°C for 90 s (25 cycles); maintained at 4°C. The sequencing reaction system was purified with 85%, 70% ethanol and deionized formamide, denatured at 96°C for 2 min, cooled to 4°C, and denatured before sequencing (ABI 3730, Thermo Fisher Scientific, USA).

#### 2.2.3 Constructing SNP haplotypes

DNA samples of couples, normal offspring, parents, other family members or probands were used to establish SNP haplotypes at the pathogenic mutation loci of the *HBA* gene and to identify valid SNP loci. To determine the *HBA* gene SNP haplotype, SNP markers within 1 Mb upstream and downstream of the related mutation loci were selected as linkage analysis markers. The target PCR products of the SNP library were enriched using the Universal NGS Library Preparation Kit (Yikon Genomics, Shanghai, China). The key steps included multiple amplification of gene-specific primers, purification of amplified products, enzymatic hydrolysis of non-target fragments, purification of enzymatic hydrolysis products, PCR enrichment library construction, product purification, and pooling. The SNP library was sequenced on the Illumina MiSeq platform (Illumina, USA). The specific SNP loci selected were analyzed by Illumina SNP Genotyping Bead Array to construct haplotypes for linkage analysis. A series of associated SNP loci upstream and downstream of the target regions were selected as genetic markers. If the father is heterozygous C/G in an SNP marker, the mother is homozygous C/C and the affected male proband inherits the mutant allele C, we can infer that the C base from the father is linked to the mutant allele and the G base is linked to the normal allele (15). The SNP haplotypes results were verified by Sanger sequencing or Gap-PCR.

#### 2.2.4 cases grouping

The grouping conditions are as follows: (i) There are more than 2 valid SNPs in the upstream and downstream regions of the *HBA* gene in the couples. The MALBAC method was selected is used for WGA. (ii) There are fewer than 2 valid SNP loci in the upstream and downstream regions of the *HBA* gene in one or both of the prospective parents. The MDA method was selected is used for WGA.

### 2.3 Blastocyst trophectoderm cells biopsy

The women who entered the PGT cycle underwent different ovulation schemes according to their situation, followed by intracytoplasmic sperm injection (ICSI) and embryo culture. Blastocyst trophoblastic ectodermal cell biopsy were obtained from blastocysts using the following protocol: (i) The embryonic biological samples for biopsy were assigned a unique number; (ii) The D3 embryos were transferred into the pre-prepared blastocyst growth dish for zona pellucida laser drilling; (iii) blastocysts ≥3 BC were selected for biopsy. The blastocysts (1/droplet) were then transferred into a biopsy dish before 5–8 trophoblast ectodermal cells were removed with a biopsy needle. Early-stage blastocysts with no trophectoderm cells observed exiting from the pore were observed for dilation at D5 PM or D6 PM. If the blastocyst had dilated but no incubated embryo cells were observed, the zona pellucida was re-drilled, and the biopsy needle was replaced to collect ectoderm cells. At the same time, a laser was used to remove the cells at the cell junction to extract the ectoderm cells; (iv) Using a biopsy needle, the biopsied blastocysts were quickly transferred into the dishes containing pre-prepared blastocyst culture medium, incubated at 37°C under 5% CO_2_ prior to freezing operation (ensuring that the number of the blastocyst freezing label is consistent with the unique biopsy number). (v) The biopsy dish containing the trophoblast ectodermal cells of the blastocyst were then at 37°C under 5% CO_2_ prior to further treatment.

### 2.4 embryo freezing

​It is important to ensure that the number of blastocysts in each frozen cell is exactly the same as the number of blastocysts at the time of the biopsy and the number of trophectoderm cells sent for PGD diagnosis. The freezing reagents ES and VS were removed from the freezer at 2-8°C and equilibrated at room temperature for 30min. The embryo is placed in ES equilibrium for about 8 min and then transferred to VS when its shape returns to 80 - 90% of its original shape. The embryos were washed several times in the VS to remove as much of the ES solution as possible. The crypt containing the embryos was placed on a pole specially designed for embryo freezing and placed in liquid nitrogen after loading. Ensure that the time between embryo washing from VS to liquid nitrogen placement is less than 1 minute. An external cannula was placed to insulate the embryo from liquid nitrogen.

### 2.5 Embryonic *HBA* gene mutation detection

#### 2.5.1 WGA

##### 2.5.1.1 MDA assay

Biopsy samples were amplified using a REPLI-g Single Cell Kit (QIAGEN, Germany) following the manufacturer’s protocol. The denaturation buffer was prepared by mixing 0.25 μL DTT (1 M) with 2.75 μL buffer DLB. Subsequently, 3 μL freshly prepared denaturing buffer was added to the sample tube containing 4 μL PBS and the biopsy sample. After incubation for 10 min at 65°C, 3 μL stop solution was added to the sample tube. The amplification mixture consisted of 9 μL H_2_O, 29 μL REPLI-g sc Reaction Buffer, and 2 μL REPLI-g sc DNA polymerase. The amplification mixture was added to 10 μL denatured DNA and incubated at 30°C for 4 h followed by heat inactivation at 85°C for 5 min.

##### 2.5.1.2 MALBAC assay

Biopsy samples were amplified using a Universal Sample Preparation Kit (Yikon Genomics, Shanghai, China). The protocol involves a cell lysis procedure to release the DNA for use as an initial template in a 5 μL reaction volume (0.5 μL lyase, 4.5 μL lysis buffer with sample). The PCR reaction conditions were as follows: 20 min at 50°C followed by 10 min at 80°C and then maintained at 4°C. The amplification mixture contained 60 μL amplification buffer solution, 2 μL amplification enzyme. Then, 60 μL of the freshly prepared amplification reaction solution was added to the lysate, and the amplification reaction was performed as follows: 94°C for 3 min; 10°C for 20 s, 30°C for 30 s, 50°C for 40 s, 70°C for 2 min, 95°C for 20 s (8 cycles); 94°C for 3 s; 94°C for 30 s, 58°C for 15 s, 72°C for 2 min (17 cycles); 72°C for 5 min; maintained at 4°C. The amplified product was immediately used in experiments or stored at −20°C.

#### 2.5.2 Gap-PCR

Gap-PCR experiments as previously described in Mutation loci detection of *HBA* gene.

#### 2.5.3 Sanger sequencing

Sanger sequencing as previously described in Mutation loci detection of *HBA* gene.

### 2.6 SNP linkage analysis for PGT-M and NGS-based analysis for PGT-A

#### 2.6.1 Construction of SNP library

Construction of SNP library as previously described Constructing SNP haplotypes.

#### 2.6.2 Construction of CNV library

The CNV library fragmentation products were enriched using the Universal DNA Fragmentation Kit (Yikon Genomics, Shanghai, China), and library construction was performed directly. The key steps involved random splicing of double-stranded DNA into 200–500 bp fragments, DNA end repair, adapter ligation, ligation product purification, PCR enrichment, and library purification. CNV libraries were sequenced on the Illumina MiSeq platform (Illumina, USA). BlueFuse Multi software was used to process and analyze the MiSeq data. Similar but not identical reads were required to pass a series of quality assurance metrics. Each aligner read count was assigned to a bin cell with a default length of 1 Mb to calculate the CNVs. Unique mapped reads were then calculated to obtain a reference data set representing the relative copy number (16, 17). Aneuploid calling was carried out manually by technicians using BlueFuse Multi based on individual observations of the deviation from the default copy number of two. The coefficient of variation (CV) was calculated as the ratio of the standard deviation of the original data to the mean of the original data. The CV value represents the quality control index used to evaluate the degree of CNV dispersion, and should be <0.25.

### 2.7 Embryo transfer

Unaffected and euploid blastocysts were identified as transferrable embryos. In the subsequent *in vitro* fertilization-embryo transfer (IVF-ET), the transplantation period was selected based on the luteal phase support. ​The specific process mainly involves the body position and vaginal cervical preparation, transfer of the embryo into the transfer tube, and transfer of the embryo into the uterine cavity. Growth fluid, air bubble, growth fluid, embryo, air bubble and growth fluid are inhaled in turn in the transplant tube. ​The implantation tube containing the embryo is inserted accurately and gently into the uterine cavity through the cervix. The growth fluid and embryo are slowly injected at a distance of 0.5 cm from the uterine floor, for a total injection of no more than 0.03 ml. The transplant tube was withdrawn after 10 seconds of standing. ​Transplant tubes and petri dishes were examined under a solid microscope to verify that embryo had been transplanted into the uterus.

### 2.8 Statistical analysis

Statistical analyses were conducted using the Mann–Whitney U-test and chi-square test in IBM SPSS Statistics software (version 20.0). *P* < 0.05 was considered to indicate statistical significance.

## 3 Results

### 3.1 Pedigree validation before PGT

The MALBAC group consisted of 72 blastocyst biopsy samples obtained from 16 couples and the MDA group consisted of 181 blastocyst biopsy samples obtained from 36 couples. The genotypes of these patients are shown in the **Supplementary Materials Excel 1**. The average maternal ages of the MALBAC and MDA groups were 33.19 ± 5.089 years and 32.50 ± 4.789 years, respectively, with no significant difference between the two groups (*P* = 0.766).

### 3.2 Comparison of the MALBAC and MDA groups

#### 3.2.1 WGA results between MALBAC and MDA groups

The average allele drop-out (ADO) rate in the MALBAC group was significantly higher than that in the MDA group (2.27% ± 3.57% *vs.* 0.97% ± 1.4%, respectively; *P* = 0.451). The results of the study were summarized below: (i) In the MALBAC group, the WGA success rate was 98.61% (71/72), WGA amplification failed in 1 sample, and the SNP detection success rate was 94.44% (68/72). The results of SNP haplotype analysis in 36 (36/72, 50%) samples were consistent with the Gap-PCR results. In the remaining 36 samples, the results of SNP haplotype detection were inconsistent with the results of Gap-PCR detection (5 samples of ADO, 27 samples of external region primer amplification failure, 2 samples of SNP detection failure due to trisomy 16/16p abnormality, and 2 samples of external region primer amplification failure with trisomy 16/16p abnormality). (ii) In the MDA group, the WGA success rate was 98.89% (179/181), WGA amplification failed in 2 samples, and the SNP detection success rate was 96.68% (175/181). The results of SNP haplotype analysis in 151 (151/181, 83.43%) samples were consistent with Sanger sequencing and/or the Gap-PCR results. In the remaining 28 samples, the results of SNP haplotype detection were inconsistent with the results of Gap-PCR detection (2 samples of ADO, 16 samples of external region primer amplification failure, 8 samples of SNP detection failure due to trisomy 16/monosomy 16/16p abnormality, and 1 sample of abnormal detection (unqualified quality control), 1 sample of triploidy). (iii) ​The chi-squared values of WGA success rate, SNP success rate, and the results of SNP and Gap-PCR/Sanger Sequencing were consistent in groups MALBAC and MDA at 0.851, 0.409 and 0, respectively. (iv) The number of valid SNP loci between groups MALBAC and MDA is statistically significant (30 ± 9 *vs.* 34 ± 10, *P*=0.02). The above data was shown **Table 1**.

**Table 1:** The results of WGA and SNP haplotype analysis in MALBAC and MDA groups.

Group	Average age of women	ADO rate	WGA success rate	SNP detection success rate	Comparison of SNP and Gap-PCR/sanger sequencing detection results		Valid SNP loci	CV value	Average number of raw reads	Coverage of genome (%)	CNVs
					Consistently	Inconsistently					
MALBAC	33.19 ± 5.089	2.27% ± 3.57%	98.61%	94.44%	50%	50% (36/72) (1) ADO: 5 (2) External region primer amplification's failure: 27 (3) Trisomy 16/Abnormal 16p: 2 (4) External region primer amplification's failure/ Abnormal 16p: 2	30 ±9	0.12 ± 0.263	3244259 ± 999124	5.02 ± 1.09	38 (38/72, 52.78%)
MDA	32.50 ± 4.789	0.97% ± 1.4%	98.89%	96.68%	83.43%	16.57% (30/181) (1) ADO: 2 (2) External region primer amplification's failure: 16 (3) Trisomy 16/Monosomy 16/Abnormal 16p: 8 (4) Abnormal detection (unqualified quality control): 1 (5) Triploid: 1	34 ±10	0.09 ± 0.40	3713146 ± 1028721	5.55 ± 1.49	86 (86/181, 47.51%)
*P* value	0.766	0.451	0.851	0.409	0		0.02	0.082	0	0.008	*P*=0.450

#### 3.2.2 The results of SNP linkage analysis were inconsistent with those of PCR-based mutation loci detection

**Figure 1:**
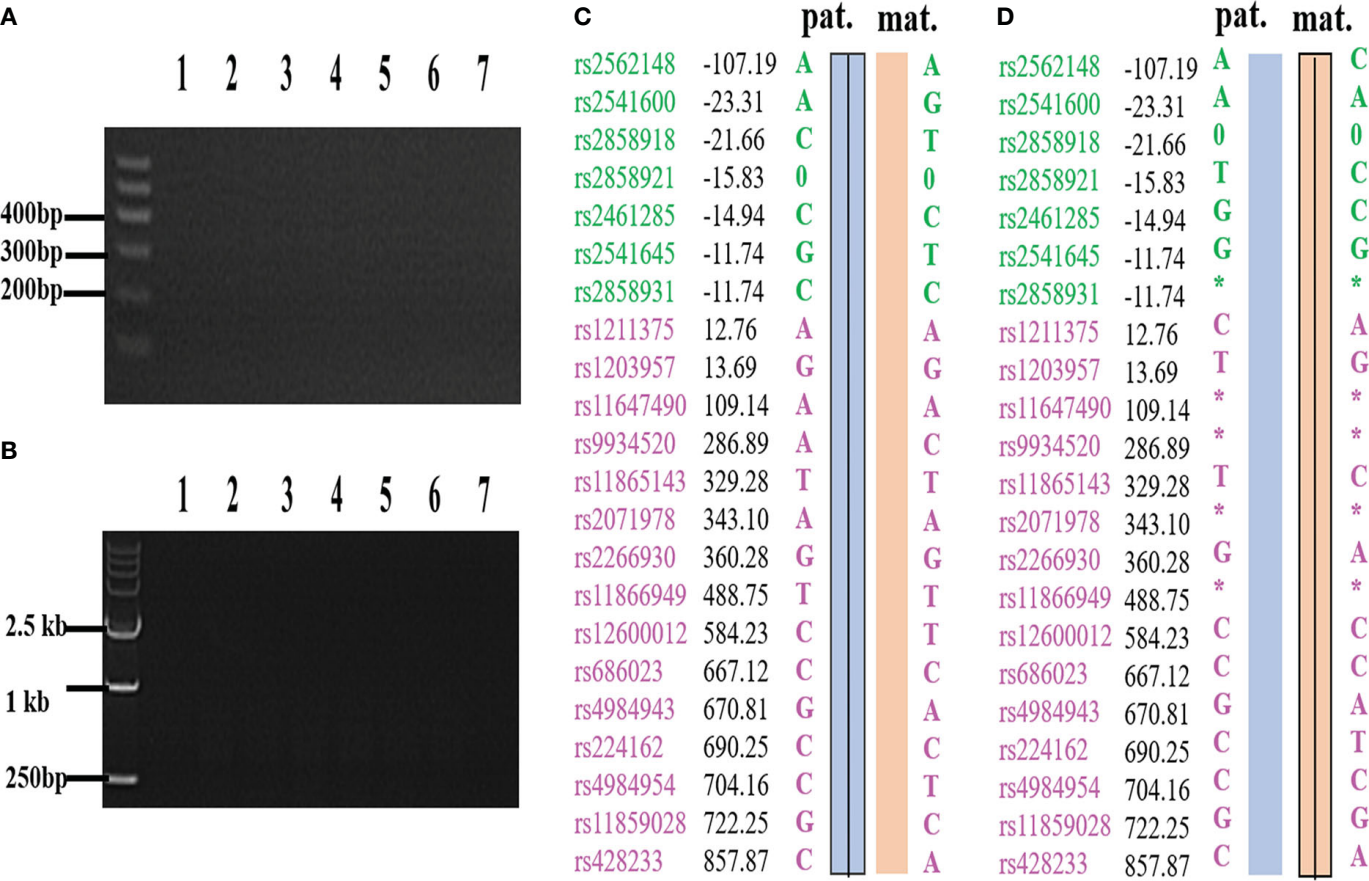
The results of PCR loci mutation detection and SNP haplotype analysis were inconsistent due to ADO and failure of external primer amplification. **(A)** –^SEA^ external primer failed to amplify in Gap-PCR assay. **(B)** -α^3.7^ external primer failed to amplify in Gap-PCR assay. **(C)** C and A are derived from the same biopsy sample, and C is the result of SNP haplotype analysis of the sample; **(D)** D and B are derived from the same biopsy sample, and D is the result of SNP haplotype analysis of the sample. Lane 1 and lane 7 were normal control and blank control, respectively. Lanes 2 to 6 are the bands of embryo samples 1 to 5 of the family, respectively. Pat: paternal haplotype, mat: maternal haplotype; *: ADO; Number: relative distance from the upstream and downstream of the *HBA* gene (unit Kb); Left SNP site: green and purple represent the SNP site upstream or downstream of the chromosomal location where the gene is located, respectively; Line within haplotype: represents pathogenic chain.

**Figure 2:**
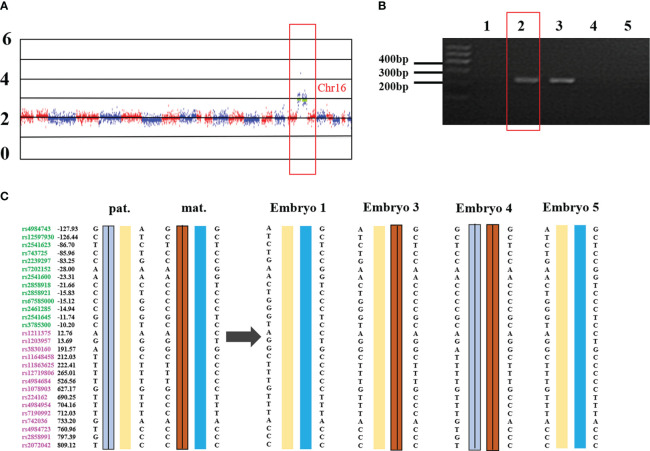
Trisomy 16 results in the failure of SNP detection in –^SEA^ thalassemia biopsy samples. **(A)** the whole genome CNV map of embryo 2. Chr: chromosome. Numbers on the left: 0, deletion; 2, diploid; 4/6: duplication. **(B)** –^SEA^ external primer amplifies in Gap-PCR assay. The red box is the electrophoresis result of embryo 2. Lane 1 and lane 7 were normal control and blank control, respectively. Lanes 2 to 6 are the bands of embryo samples 1 to 5 of the family, respectively. **(C)** SNP linkage analysis. Pat: Paternal, mat: maternal; Number: relative distance from the upstream and downstream of the *HBA* gene (unit Kb); Left SNP site: green and purple represent the SNP site upstream or downstream of the chromosomal location where the gene is located, respectively; Line within haplotype: represents pathogenic chain.

External region primer amplification failure, Sanger sequencing failure, and a high ADO rate can lead to inconsistent results between the SNP analysis and the PCR-based mutation loci detection. If the SNP linkage analysis is inconsistent with the Sanger sequencing and/or Gap-PCR results, the results of the SNP linkage analysis prevail. If the SNP linkage analysis fails, the Sanger sequencing and/or Gap-PCR results are accepted. In addition, CNVs in the *HBA* gene region of chromosome 16 will lead to failure in constructing the SNP haplotype. At this time, the diagnosis of pathogenic loci in embryos is based on PCR-based mutation loci detection. The detection results for some embryos indicated that the embryos did not carry the *HBA* gene mutation, while SNP linkage analysis showed that these embryos did. For example, the MALBAC method was used for WGA when the genotypes of a couple were identified as –^SEA^/αα and -α^3.7^/αα. SNP analysis revealed that the couple’s affected two embryos were α-thalassemia minor (–^SEA^/αα) and a silent gene (-α^3.7^/αα) carrier. However, corresponding electrophoretic bands were not detected in the Gap-PCR analysis of the two affected embryos due to the failure of the external primer amplification for –^SEA^ and -α^3.7^ (**Figure 1**). SNP assay failed in samples with trisomy on chromosome 16 or CNV of chromosome 16p. **Figure 2** shows abnormal SNP detection in embryo 2, and the corresponding band appeared in the second lane of the Gap-PCR analysis, indicating that the embryos were unaffected.

### 3.3 PGT-A outcomes

**Figure 3:**
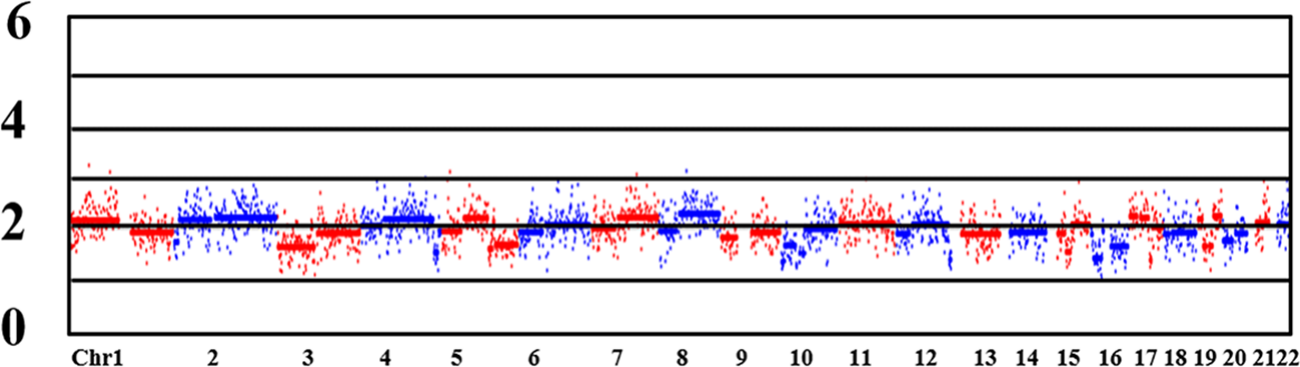
CNV map of the whole genome. Chr: chromosome. Numbers on the left: 0, deletion; 2, diploid; 4/6: duplication. Lane 1 and lane 7 were normal control and blank control, respectively. Lanes 2 to 6 are the bands of embryo samples 1 to 5 of the family, respectively.

CNV detection is used to identify fragments with deletion/duplication >4 Mb, and mosaic >40% of chromosome. In the study, the CNV status of the MALBAC and MDA groups were confirmed by NGS-based PGT-A. ​Three samples in each group failed the CNV analysis, as did the *HBA* allele amplification, confirming the WGA failure. The MALBAC and MDA groups had 38 (38/72, 52.78%) and 86 (86/181, 47.51%) embryos with CNVs, respectively (*P*=0.450). In some cases, we found that CV values of two embryos in the MALBAC group were <0.25, and there were no abnormal fragments larger than 4 Mb. But, the overall genome-wide CNV map showed that the CNVs were scattered rather than concentrated (**Figure 3**). ​The basic quality control parameters for the two groups of sequencing results are as follows: (i) The mean CV values of MALBAC and MDA groups are 0.09 ± 0.40 and 0.12 ± 0.263, respectively. ​The CV values were not statistically significant in the two groups (*P*=0.082). (ii) The average number of raw reads was 3244259 ± 999124 and 3713146 ± 1028721 for group MALBAC and MDA, respectively. There was a statistically significant difference between the two groups (*P*=0). (iii) The coverage of genome (%), MALBAC *vs.* MDA = 5.02 ± 1.09 *vs.* 5.55 ± 1.49. ​The difference between the two groups was statistically significant (*P*=0.008).

### 3.4 Clinical outcomes

The optimal embryos were selected for transplantation based on the results of genetic testing and embryo quality analysis. Among the 38 cases of IVF-ET, successful implantation occurred in 32 cases, 13 cases are ongoing pregnancy, biochemical pregnancy occurred in 8 cases, successful delivery was achieved in 11 cases. **Table 2** describes the healthy live births in 11 different families after PGT-M and PGT-A treatment. The 11 families chose single embryo transfer, no chromosomal abnormalities >4 Mb were identified. Invasive prenatal diagnosis was performed through amniocentesis at 12 weeks of pregnancy. The results of prenatal diagnosis were consistent with those of the pre-implantation genetic testing.

**Table 2:** The primary clinical information of the 11 healthy live births.

Family	Maternal genotype	Paternal genotype	Maternal age (year)	Embryo genotype	Embryo grading	WGA method	Embryo Karyotype
5	--^SEA^/αα	--^SEA^/αα	37	αα/αα	4AA	MALBAC	46,XN
6	--^SEA^/αα	-α^3.7^/αα	30	αα/αα	4AB	MALBAC	46,XN
7	--^SEA^/αα	--^SEA^/αα	29	αα/αα	4AA	MALBAC	46,XN
9	-α^4.2^/αα	--^SEA^/αα	28	αα/αα	4AA	MALBAC	46,XN
16	-α^3.7^/αα	--^SEA^/αα	33	αα/αα	4BB	MALBAC	46,XN
19	--^SEA^/α^WS^α	--^SEA^/αα	29	α^WS^α/αα	4AA	MDA	46,XN
25	--^SEA^/αα	-α^4.2^/αα	39	αα/αα	4AA	MDA	46,XN
29	--^SEA^/αα	--^SEA^/-α^3.7^	32	-α^3.7^/αα	4AB	MDA	46,XN
32	--^SEA^/αα	--^SEA^/αα	30	αα/αα	4AA	MDA	46,XN
33	-α^3.7^/αα	--^SEA^/αα	31	αα/αα	4AB	MDA	46,XN
34	--^SEA^/αα	--^SEA^/αα	33	--^SEA^/αα	4BB	MDA	46,XN

## 4 Discussion

Since the *HBA* gene is located near the telomere (comprises only 200 kb). Consequently, there are fewer SNPs upstream of the *HBA* gene. The distribution of SNP locus in the human genome is homogeneous with a small spatial extent (18). Sufficient SNP loci available upstream and downstream for both male and female partners (SNPs >2 or more) can prevent misinterpretation caused by a low proportion of chromosomal exchanges. In MALBAC group (SNPs≥2), SNP haplotype analysis does not depend on Gap-PCR results to determine the pathogenic loci of samples, and can avoid the risk of misinterpretation due to chromosome exchange. In MDA group (SNP<2), the determination of the pathogenic loci in the samples using SNP haplotype analysis require combination of Gap-PCR results. ​If no upstream SNP locus is available, the risk of misjudgment due to chromosome exchange cannot be avoided. What’s more, external primer amplicons of large fragment deletion may not be possible direct detection if MALBAC method (amplification fragment length: 0.2-2kb) is used.

Our data showed that the ADO rate of the MDA group was lower than that of the MALBAC group (0.97% ± 1.4% *vs.* 2.27% ± 3.57%, *P*=0.451) and the consistency rate between Sanger sequencing and/or Gap-PCR results and SNP haplotype analysis in MDA group was higher than that in the MALBAC group (83.43% *vs*. 50%, *P*=0). As a foundation for PGT, the WGA should be selected based on clinical application (19). ​ MDA is a non-PCR-based amplification technique that uses multiple displacement Phi29 DNA polymerase with random primers under isothermal conditions to amplify the target genome (20). Compared with PCR-based WGA methods, MDA reduces amplification bias by 4–6 orders of magnitude, and produces fragments of >10 kb (21). In contrast, MALBAC produces short fragments, rendering this method more prone to allele deletion (22). Thus, MDA can avoid false negatives due to amplification. Moreover, the MDA group had a higher number of valid SNPs than the MALBAC group in the study. It was found that the amplification efficiency of the MDA technique is higher than that of the MALBAC technique, and that the SNP is better detected (22).

Our results demonstrate that SNP haplotype analysis combined with Gap-PCR and Sanger sequencing could improve the diagnosis of whether embryos from patients with deletional α-thalassemia carry the *HBA* gene mutation. ​Gap-PCR cannot detect both deletions and mutations and requires manual interpretation of the electrophoretic maps, which is not only prone to human error but also difficult to improve efficiency. SNP haplotype analysis only requires the establishment of a detection process to detect both deletions and mutations, and the procedure is simple and easy to automate. SNP loci can be used to accurately determine the parental origin of the target genes, especially chromosome recombination and localization (18). There are unaffected relatives in the family, haplotype analysis can still be carried out by directly detecting the mutation loci by NGS and using the affected embryos or gametes as probands (23). However, the deletion regions of the -α^3.7^ and -α^4.2^ were not fixed and were in a floating state, resulting in large primer amplified fragments. While the sequencing fragments of the NGS platform were short, and it was difficult to detect the entire PCR amplified fragments. ​In addition, SNP haplotype analysis cannot be performed when CNV is present in the *HBA* gene on chromosome 16. ​In summary, it is necessary to detect *HBA* gene through both SNP and Gap-PCR/Sanger sequencing.

There was no significant difference in CV values between the MDA and MALBAC groups in our study. Moreover, the average number of raw reads and the coverage of genome (%) of MDA group are superior to that of MALBAC group. This suggests that the MDA method is also suitable for CNV analysis in deletional α-thalassemia. The finding was in contrast to previous study, where MALBAC yielded the most uniform CNV values after normalization (19). We speculated that this discrepancy might be related to differences in the developmental stages of the embryos analyzed. We focused mainly on blastocyst trophectoderm cells from deletional α-thalassemia, while the previous study focused mainly on fibroblast samples with defined β-thalassemia variations and single-blastomere samples. Although our data showed no abnormal CNV fragments >4 Mb in several samples, the overall genome-wide CNV map was relatively scattered. However, the risk of abnormalities in such embryos with CNV smaller than 4 Mb remains. Therefore, we recommend a second biopsy to confirm the presence of small pathogenic CNV fragments of in the samples. Without a second biopsy, these embryos should be inspected carefully before transplantation. There is also a special case where all embryo samples from the same patient have deletions or duplications in similar regions. The abnormal fragments in some embryos may occur in two segments, especially the 46, XN embryos reported at 4 M resolution. This observation should be considered from two perspectives: (i) The CNVs may come from parents. In this case, we recommend that the couples get tested for genome-wide CNV to confirm whether the CNVs are inherited; (ii) We should also determine whether the CNVs have been recorded in genetic databases such as OMIM and DECIPHER.

PGT-M plus PGT-A cycles have resulted in a significantly increased pregnancy rate and decreased spontaneous abortion rates compared to PGT-M processes alone (24). Even in younger women, PGT-M plus PGT-A significantly improved live birth rates of FET cycles compared to PGT-M alone (25). Since the rate of chromosomal abnormalities increases with maternal age (26), the cost-effectiveness of PGT-A would increase with maternal age (27). PGT-M combined with PGT-A provides 80% of couples with satisfactory genetic test results (28). Thus, we recommend that deletional α-thalassemia patients undergoing PGT-assisted conception may consider screening of unaffected or heterozygous embryos for aneuploidy to achieve higher implantation and pregnancy rates.

## 5 Conclusions

Although the limitation of this study is that the number of deletional α-thalassemia samples is small, we found that MDA is superior to MALBAC as a method for establishing a PGT system for deletional α-thalassemia. SNP haplotype analysis and PCR mutation loci detection were applied for PGT-M diagnosis of pathogenic genes to improve the clinical diagnosis rate and avoid false-negative/false-positive results. ​Based on the WGA, the combination of PGT-M and PGT-A has been shown to improve clinical pregnancy outcomes in patients with deletional α-thalassemia.

## Data availability statement

The original contributions presented in the study are included in the article/**Supplementary Material**. Further inquiries can be directed to the corresponding authors.

## Ethics statement

The studies involving human participants were reviewed and approved by the Ethics Committee of Maternal and Child Health Hospital of Guangxi Zhuang Autonomous Region. Written informed consent to participate in this study was provided by the participants’ legal guardian/next of kin. Written informed consent was obtained from the individual(s), and minor(s)’ legal guardian/next of kin, for the publication of any potentially identifiable images or data included in this article.

## Author contributions

Conducting experiments: YL, LL, and PH. Design experiment: LY, PH, and HZ. Collecting patient information: YL. Embryo biopsy: JS, CW, XZ. Literature search and data analysis: YL, PH. Writing—original draft preparation: YL. Writing—review and editing: PH, YL, and QQ. Critical revision for important intellectual content: JL, SH, and HW. All authors have read and agreed to the published version of the article.

## Supplementary material

Supplementary Table 1 The primers sequence.

Supplementary Material Excel 1 Genotypes of patients and embryos.

## References

[1] Chen D Shen X Wu C Xu Y Ding C Zhang G. Eleven healthy live births: a result of simultaneous preimplantation genetic testing of α- and β-double thalassemia and aneuploidy screening. J Assist Reprod Genet (2020) 37(3):549–57. doi: 10.1007/s10815-020-01732-7 PMC7125281 32152910

[2] Merkeley H Bolster L. Thalassemia. Cmaj (2020) 192(41):E1210. doi: 10.1503/cmaj.191613 33051316 PMC7588257

[3] Farashi S Harteveld CL. Molecular basis of α-thalassemia. Blood Cells Mol Dis (2018) 70:43–53. doi: 10.1016/j.bcmd.2017.09.004 29032940

[4] Lai K Huang G Su L He Y. The prevalence of thalassemia in mainland China: evidence from epidemiological surveys. Sci Rep (2017) 7(1):920. doi: 10.1038/s41598-017-00967-2 28424478 PMC5430438

[5] Jomoui W Fucharoen G Sanchaisuriya K Charoenwijitkul P Maneesarn J Xu X. Genetic origin of α(0)-thalassemia (SEA deletion) in Southeast Asian populations and application to accurate prenatal diagnosis of Hb Bart's hydrops fetalis syndrome. J Hum Genet (2017) 62(8):747–54. doi: 10.1038/jhg.2017.41 PMC5584512 28381876

[6] Singer ST. Variable clinical phenotypes of alpha-thalassemia syndromes. Sci World J (2009) 9:615–25. doi: 10.1100/tsw.2009.69 PMC5823233 19618088

[7] Lai K Li S Lin W Yang D Chen W Li M. Invasive prenatal diagnosis of α-thalassemia to control Hb Bart's hydrops fetalis syndrome: 15 years of experience. Arch Gynecol Obstet (2018) 298(2):307–11. doi: 10.1007/s00404-018-4807-4 29948167

[8] King AJ Higgs DR. Potential new approaches to the management of the Hb Bart's hydrops fetalis syndrome: the most severe form of α-thalassemia. Hematol Am Soc Hematol Educ Program (2018) 2018(1):353–60. doi: 10.1182/asheducation-2018.1.353 PMC6246003 30504332

[9] Li Y Liang L Tian M Qing T Wu X. Electrophoresis features and genotypes of Hb bart's hydrops fetalis. Scand J Clin Lab Invest (2020) 80(2):129–32. doi: 10.1080/00365513.2019.1703211 31841045

[10] Zegers-Hochschild F Adamson GD Dyer S Racowsky C de Mouzon J Sokol R. The international glossary on infertility and fertility care, 2017. Fertil Steril (2017) 108(3):393–406. doi: 10.1016/j.fertnstert.2017.06.005 28760517

[11] Carvalho F Moutou C Dimitriadou E Dreesen J Giménez C Goossens V. ESHRE PGT Consortium good practice recommendations for the detection of monogenic disorders. Hum Reprod Open (2020) 2020(3):hoaa018. doi: 10.1093/hropen/hoaa018 32500103 PMC7257022

[12] Richards S Aziz N Bale S Bick D Das S Gastier-Foster J. Standards and guidelines for the interpretation of sequence variants: a joint consensus recommendation of the American College of Medical Genetics and Genomics and the Association for Molecular Pathology. Genet Med (2015) 17(5):405–24. doi: 10.1038/gim.2015.30 PMC4544753 25741868

[13] Ou Z Deng Y Liang Y Chen Z Sun L. Using affected embryos to establish linkage phase in preimplantation genetic testing for thalassemia. Reprod Biol Endocrinol (2022) 20(1):75. doi: 10.1186/s12958-022-00948-9 35490243 PMC9055750

[14] Parikh FR Athalye AS Naik NJ Naik DJ Sanap RR Madon PF. Preimplantation genetic testing: its evolution, where are we today? J Hum Reprod Sci (2018) 11(4):306–14. doi: 10.4103/jhrs.JHRS_132_18 PMC6333033 30787513

[15] Wang Y Zhu X Yan Z Zhi X Guan S Kuo Y. Novel PGD strategy based on single sperm linkage analysis for carriers of single gene pathogenic variant and chromosome reciprocal translocation. J Assist Reprod Genet (2020) 37(5):1239–50. doi: 10.1007/s10815-020-01753-2 PMC7244654 32350783

[16] Chuang TH Wu ZH Kuan CS Lee MJ Hsieh CL Wang HL. High concordance in preimplantation genetic testing for aneuploidy between automatic identification via Ion S5 and manual identification via Miseq. Sci Rep (2021) 11(1):18931. doi: 10.1038/s41598-021-98318-9 34556730 PMC8460708

[17] Ji X Zhang Z Shi J He B. Clinical application of NGS-based SNP haplotyping for the preimplantation genetic diagnosis of primary open angle glaucoma. Syst Biol Reprod Med (2019) 65(3):258–63. doi: 10.1080/19396368.2019.1590479 30977407

[18] Wang J Lu BM Li R Guo J Xu Y Pan JF. Karyomapping in preimplantation genetic testing for β-thalassemia combined with HLA matching: a systematic summary. J Assist Reprod Genet (2019) 36(12):2515–23. doi: 10.1007/s10815-019-01595-7 PMC6911139 31758512

[19] Liu W Zhang H Hu D Lu S Sun X. The performance of MALBAC and MDA methods in the identification of concurrent mutations and aneuploidy screening to diagnose beta-thalassaemia disorders at the single- and multiple-cell levels. J Clin Lab Anal (2018) 32(2):e22267. doi: 10.1002/jcla.22267 28548214 PMC6817139

[20] Ning L Li Z Wang G Hu W Hou Q Tong Y. Quantitative assessment of single-cell whole genome amplification methods for detecting copy number variation using hippocampal neurons. Sci Rep (2015) 5:11415. doi: 10.1038/srep11415 26091148 PMC4650676

[21] Dean FB Hosono S Fang L Wu X Faruqi AF Bray-Ward P. Comprehensive human genome amplification using multiple displacement amplification. Proc Natl Acad Sci U.S.A. (2002) 99(8):5261–6. doi: 10.1073/pnas.082089499 PMC122757 11959976

[22] He F Zhou W Cai R Yan T Xu X. Systematic assessment of the performance of whole-genome amplification for SNP/CNV detection and β-thalassemia genotyping. J Hum Genet (2018) 63(4):407–16. doi: 10.1038/s10038-018-0411-5 29440707

[23] Ren Y Zhi X Zhu X Huang J Lian Y Li R. Clinical applications of MARSALA for preimplantation genetic diagnosis of spinal muscular atrophy. J Genet Genomics (2016) 43(9):541–7. doi: 10.1016/j.jgg.2016.03.011 27599922

[24] Rechitsky S Pakhalchuk T San Ramos G Goodman A Zlatopolsky Z Kuliev A. First systematic experience of preimplantation genetic diagnosis for single-gene disorders, and/or preimplantation human leukocyte antigen typing, combined with 24-chromosome aneuploidy testing. Fertil Steril (2015) 103(2):503–12. doi: 10.1016/j.fertnstert.2014.11.007 25516085

[25] Hou W Xu Y Li R Song J Wang J Zeng Y. Role of aneuploidy screening in preimplantation genetic testing for monogenic diseases in young women. Fertil Steril (2019) 111(5):928–35. doi: 10.1016/j.fertnstert.2019.01.017 30922652

[26] Munné S Alikani M Ribustello L Colls P Martínez-Ortiz PA McCulloh DH. Euploidy rates in donor egg cycles significantly differ between fertility centers. Hum Reprod (2017) 32(4):743–9. doi: 10.1093/humrep/dex031 28333245

[27] Facadio Antero M Singh B Pradhan A Gornet M Kearns WG Baker V. Cost-effectiveness of preimplantation genetic testing for aneuploidy for fresh donor oocyte cycles. F S Rep (2021) 2(1):36–42. doi: 10.1016/j.xfre.2020.11.005 34223271 PMC8244284

[28] Satirapod C Sukprasert M Panthan B Charoenyingwattana A Chitayanan P Chantratita W. Clinical utility of combined preimplantation genetic testing methods in couples at risk of passing on beta thalassemia/hemoglobin E disease: A retrospective review from a single center. PloS One (2019) 14(11):e0225457. doi: 10.1371/journal.pone.0225457 31751397 PMC6872132

