# Supplementary material for: Appropriate whole genome amplification and pathogenic loci detection can improve the accuracy of preimplantation genetic diagnosis for deletional α-thalassemia
Source: Front Endocrinol (Lausanne). 2024 Mar 8;14:1176063. doi: 10.3389/fendo.2023.1176063 (PMC10957767; doi:10.3389/fendo.2023.1176063)
Supplement: Supplementary Material Excel 1 — Genotypes of patients and embryos. [file Table_2.docx]

| locus | Deletion region external primer | Standard internal control primer |
| --- | --- | --- |
| --^SEA^ | F-GGGCTCTGTGTTCTCAGTATT | F-GTCTCACCTCAATCATCCTGTG |
|  | R-TGCTTTGTCACCCATGCT | R-CACCTCTGGGTAGGTTCTGTA |
| -α^4.2^ | F-GTTTACCCATGTGGTGCCTC | F-ATCTCCTGACCTCGCATCT |
|  | R-CCCGTTGGATCTTCTCATTTCCC | R-ACTCTAGCCTGACGACACA |
| -α^3.7^ | F-CCCCTCGCCAAGTCCACCC | F-CCTTGTCTCCTCTGTCCTTTC |
|  | R-AAAGCACTCTAGGGTCCAGCG | R-GGAGGTAGGCAGTCCTCTAA |
| α^WS^ | F- GCCGCACTGACCCTCTTCTCTG and R- CGGGCAGGAGGAACGGCTAC | |

​
